# Supplementary material for: Phosphatidic acid signaling in modulating plant reproduction and architecture
Source: Plant Commun. 2024 Dec 24;6(2):101234. doi: 10.1016/j.xplc.2024.101234 (PMC11897466; doi:10.1016/j.xplc.2024.101234)
Supplement: Document S1. Supplemental Table 1 [file mmc1.pdf]

Plant Communications, Volume *6*

Supplemental information

Phosphatidic acid signaling in modulating plant reproduction and architecture

Shuaibing Yao, Bao Yang, Jianwu Li, Shan Tang, Shaohua Tang, Sang-Chul Kim, and Xuemin Wang

Supplemental Table 1. Accession numbers of genes encoding PA production and removal enzymes in Arabidopsis

Gene Family	Gene name	Accession number
Phospholipase D	PLDα1	AT3G15730
	PLDα2	AT1G52570
	PLDα3	AT5G25370
	PLDε	AT1G55180
	PLDβ1	AT2G42010
	PLDβ2	AT4G00240
	PLDγ1	AT4G11850
	PLDγ2	AT4G11830
	PLDγ3	AT4G11840
	PLDδ	AT4G35790
	PLDζ1	AT3G16785
	PLDζ2	AT3G05630
Diacylglycerol kinase	DGK1	AT5G07920
	DGK2	AT5G63770
	DGK3	AT2G18730
	DGK4	AT5G57690
	DGK5	AT2G20900
	DGK6	AT4G28130
	DGK7	AT4G30340
Lysophosphatidic acid acyltransferase	LPAT1	AT4G30580
	LPAT2	AT3G57650
	LPAT3	AT1G51260
	LPAT4	AT1G75020
	LPAT5	AT3G18850
Non-specific phospholipase C	NPC1	AT1G07230
	NPC2	AT2G26870
	NPC3	AT3G03520
	NPC4	AT3G03530
	NPC5	AT3G03540
	NPC6	AT3G48610
Phosphatidylinositol-hydrolyzing phospholipase C	PLC1	AT5G58670
	PLC2	AT3G08510
	PLC3	AT4G38530
	PLC4	AT5G58700
	PLC5	AT5G58690
	PLC7	AT3G55940
	PLC8	AT3G47290
	PLC9	AT3G47220

Phosphatidic acid phosphohydrolase	PAH1	AT3G09560
	PAH2	AT5G42870
Lipid phosphate phosphatase	LPP1	AT2G01180
	LPP2	AT1G15080
	LPP3	AT3G02600
	LPP4	AT3G18220
	LPPβ	AT4G22550
	LPPγ	AT5G03080
	LPPδ	AT3G58490
	LPPε1	AT3G50920
	LPPε2	AT5G66450
Cytidine diphosphate-diacylglycerol synthase	CDS1	AT1G62430
	CDS2	AT4G22340
	CDS3	AT4G26770
	CDS4	AT2G45150
	CDS5	AT3G60620
	CDS6	AT3G47630
