# Supplementary material for: Phosphatidic acid signaling in modulating plant reproduction and architecture
Source: Plant Commun. 2024 Dec 24;6(2):101234. doi: 10.1016/j.xplc.2024.101234 (PMC11897466; doi:10.1016/j.xplc.2024.101234)
Supplement: Document S2. Article plus supplemental information [file mmc2.pdf]

Plant Communications
Review article

CellPress Partner Journal

Phosphatidic acid signaling in modulating plant reproduction and architecture

Shuaibing Yao[1,2,4], Bao Yang[1,2,4], Jianwu Li[1,2], Shan Tang[1,2], Shaohua Tang[1,2], Sang-Chul Kim[1,2,3] and Xuemin Wang[1,2,*]

[1]Department of Biology, University of Missouri-St. Louis, St. Louis, MO 63121, USA

[2]Donald Danforth Plant Science Center, St. Louis, MO 63132, USA

[3]Present address: Plant Systems Engineering Research Center, Korea Research Institute of Bioscience and Biotechnology (KRIBB), Daejeon 34141, Republic of Korea

[4]These authors contributed equally to this article.

***Correspondence: Xuemin Wang (wangxue@umsl.edu)**

https://doi.org/10.1016/j.xplc.2024.101234

ABSTRACT

Phosphatidic acid (PA) is an important class of signaling lipids involved in various biological processes in plants. Functional characterization of mutants of PA-metabolizing enzymes, combined with lipidomics and protein–lipid interaction analyses, has revealed the key role of PA signaling in plant responses to biotic and abiotic stresses. Moreover, PA and its metabolizing enzymes influence several reproductive processes, including gametogenesis, pollen tube growth, self-incompatibility, haploid embryo formation, embryogenesis, and seed development. They also play a significant role in shaping plant reproductive and root architecture. Recent studies have shed light on the diverse mechanisms of PA's action, though much remains to be elucidated. Here, we summarize recent advances in the study of PA and its metabolizing enzymes, emphasizing their roles in plant sexual reproduction and architecture. We also explore potential mechanisms underlying PA's functions and discuss future research directions.

Key words: phosphatidic acid, phospholipases, DAG kinases, lipid signaling, plant reproduction, root architecture

Yao S., Yang B., Li J., Tang S., Tang S., Kim S.-C., and Wang X. (2025). Phosphatidic acid signaling in modulating plant reproduction and architecture. Plant Comm. **6**, 101234.

INTRODUCTION

Lipids play an essential role in membrane structure, energy metabolism, and cellular regulation. Although phosphatidic acid (PA) is a minor component of cell membranes, it serves as a central intermediate in the synthesis of membrane lipids and energy-storing triacylglycerol. The cellular levels of PA are highly dynamic in response to various stress conditions. Stress-induced production of PA plays an important role in plant responses to abiotic and biotic challenges, as highlighted in several recent reviews (Liang et al., 2023; Sharma et al., 2023; Amokrane et al., 2024; Wang et al., 2024a; Gong et al., 2024; Yao et al., 2024). In addition, the role of PA in nuclear signaling was recently reviewed (Yao et al., 2024). This review focuses on recent advances in our understanding of PA and its metabolizing enzymes in regulating plant reproduction and architecture, including pollen tube growth, self-incompatibility (SI), haploid embryo formation and seed development, root architecture, and above-ground architecture. We will also explore possible mechanisms by which PA affects these processes and discuss future research directions.

PA METABOLISM

PA is the simplest class of diacylglycerophospholipids, characterized by having only one phosphate as its head group. Cellular PA consists of various molecular species because the two fatty acyl chains can differ in their number of carbons and double bonds (Figure 1). Much of what is known about PA's function has been derived from studies on the genes and enzymes involved in PA metabolism. PA metabolism involves multiple enzymes (Figure 1; Supplemental Table 1). This intricate network regulates the production, removal, and homeostasis of cellular PA.

PA production reactions

Cellular PA is primarily produced through three reactions (Figure 1). Phospholipase D (PLD) hydrolyzes common membrane phospholipids, such as phosphatidylcholine (PC) and phosphatidylethanolamine (PE), to generate PA and a free head group. Diacylglycerol (DAG) kinase (DGK) uses ATP to phosphorylate DAG to generate PA. Lysophosphatidic acid acyltransferase (LPAAT) produces PA by acylating lysophosphatidic acid using acyl-coenzyme A, a process involved

This is an open access article under the CC BY license (http://creativecommons.org/licenses/by/4.0/).

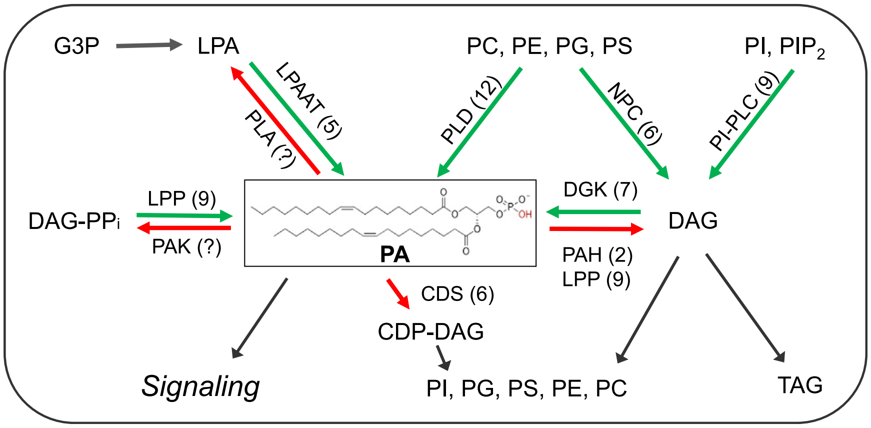

Figure 1. Metabolic network of PA production and removal.
Green and red arrows indicate the production and removal of PA, respectively. Numbers in parentheses indicate the number of genes in *Arabidopsis*. CDP-DAG, cytidine diphosphate diacylglycerol; CDS, cytidine diphosphate synthase; DAG, diacylglycerol; DAG-PPi, DAG-pyrophosphate; DGK: DAG kinase; G3P: glycerol-3-phosphate; LPA: lysophosphatidic acid; LPP: lipid phosphate phosphatase; LPAAT: LPA acyltransferase; NPC: nonspecific PLC; PA: phosphatidic acid; PAH: PA phosphohydrolases; PAK: PA kinase; PC: phosphatidylcholine; PE: phosphatidylethanolamine; PG: phosphatidylglycerol; PI: phosphatidylinositol; PIP_2: PI 4:5 bisphosphate; PLA: phospholipase A; PLC: phospholipase C; PI-PLC: PI-hydrolyzing PLC; PLD: phospholipase D; PS: phosphatidylserine; TAG: triacylglycerol.

in *de novo* glycerolipid biosynthesis, also known as the Kennedy pathway. Among these, PLD and DGK are the major enzyme families responsible for generating signaling PA. In addition, DAG, the substrate for DGK, can be produced by two families of phospholipase C (PLC): phosphatidylinositol (PI)-hydrolyzing PI-PLC and nonspecific PLC (NPC) (Wang et al., 2006).

Each of these enzymes consists of multiple members encoded by gene families (Figure 1; Supplemental Table 1). In *Arabidopsis thaliana* (*At*), for example, the PLD family has 12 members, divided into two subfamilies based on protein structure: C2-PLDs (PLDα1–3, PLDβ1–2, PLDγ1–3, PLDδ, and PLDε) and PX/PH-PLDs (PLDζ1–2). The DGK family has seven members, DGK1–7, grouped into three clades based on their conserved catalytic kinase domains and transmembrane helices (Ge et al., 2012; Yuan et al., 2019). Additionally, there are six NPCs (NPC1–6), nine PI-PLCs (PLC1–9), and five LPAATs (LPAAT1–5). Individual members of the PLD, DGK, PI-PLC, NPC, and LPAAT families often exhibit distinct functions in plant growth, development, and stress responses. For example, individual PLD enzymes are activated in unique ways depending on specific cofactors such as Ca^{2+}, lipids, or protein interactions, leading to different physiological functions (reviewed in Yao et al., 2024). Different DGK clades are involved in both biotic and abiotic stress responses (Tan et al., 2018; Vaz Dias et al., 2019; Angkawijaya et al., 2020; Tang et al., 2020; Wong et al., 2020; Li et al., 2024a; Kong et al., 2024; Qi et al., 2024). Individual NPCs are involved in various physiological processes, including responses to phosphorus deficiency and salinity stress, gametophyte and root development, and seed oil production (Cai et al., 2020b; Nakamura and Ngo, 2020; Yang et al., 2021; Li et al., 2024b; Li et al., 2025). However, genetic redundancy within these enzyme families, particularly those with similar subcellular localizations and expression patterns, complicates the elucidation of their specific roles.

PA-removal reactions

Three families of enzymes are well-documented in the removal of PA: PA phosphohydrolases (PAHs), lipid phosphate phosphatases (LPPs), and cytidine diphosphate (CDP)-DAG synthases (CDSs) (Figure 1; Supplemental Table 1). PAHs are cytosolic PA phosphatases that dephosphorylate PA into DAG, which serves as a precursor for the biosynthesis of different glycerolipids, including PC, PE, glycoglycerolipids, and triacylglycerol (Eastmond et al., 2010; Craddock et al., 2015; Nakamura, 2017). LPPs are integral membrane-associated phosphatases that dephosphorylate PA and other lipid phosphates (Su et al., 2023). CDSs are membrane-bound enzymes that catalyze the transfer of a cytidyl group from CTP to PA to form CDP-DAG, which serves as the activated precursor for the biosynthesis of PI, phosphatidylglycerol (PG), and phosphatidylserine (PS) (Figure 1) (Zhang et al., 2023).

The *Arabidopsis* genome encodes two *PAH*s, nine *LPP*s, and six *CDS*s (Figure 1; Supplemental Table 1). LPPs are further divided into two subfamilies: the "eukaryotic" type (LPPα1–α4), which is homologous to animal LPPs, and the "prokaryotic" type (LPPβ, LPPγ, LPPδ, LPPε1, and LPPε2), which is homologous to cyanobacterial LPPs (Nakamura et al., 2007; Sato and Awai, 2017; Su et al., 2023). CDSs are associated with the endoplasmic reticulum, plastids, and mitochondria (Zhang et al., 2023). In the endoplasmic reticulum, CDSs supply CDP-DAG for the biosynthesis of PG, PI, and its phosphorylated derivatives, PI phosphates (PIPs). In plastids and mitochondria, CDSs provide substrates for the production of PG, which serves as a precursor for cardiolipin biosynthesis (Zhang et al., 2023). In addition, PA can be phosphorylated by PA kinase to form DAG-pyrophosphate, thus reducing the levels of signaling PA. However, the gene encoding PA kinase has not yet been identified, and the function of DAG-pyrophosphate is largely unknown in plants (Jeannette et al., 2010).

Modulating the activity of PAHs, LPPs, and CDSs has profound effects on plant growth, development, and stress responses, often affecting the cellular levels of PA. Since these enzymes use PA as a substrate and play critical roles in lipid biosynthesis, it can be challenging to determine whether their effects result from the accumulation of PA or from the production of their lipid products, such as PI, PIPs, PG, and cardiolipin. Studies addressing this question suggest that increased PA accumulation can contribute to altered phenotypes due to changes in enzyme activity (Hong et al., 2018; Kim et al., 2019; Sha et al., 2023).

Detection of PA and its changes at the subcellular level

PA is a minor class of membrane lipids, however, its cellular levels change rapidly in response to different stress conditions (Li et al., 2019). Understanding PA's function requires the ability to detect and quantify it with spatiotemporal resolution. Several approaches have been used to analyze PA, including mass spectrometry-based lipidomic profiling, PA biosensors, and traditional lipid separation and quantification methods. Mass spectrometry allows for the identification and quantification of PA molecular species, such as the number of carbons and double bonds in the two fatty acyl chains (Welti et al., 2002). PA biosensors have been employed to monitor PA dynamics at both cellular and subcellular levels. These include membrane translocation-based single fluorescent PA biosensors and Förster resonance energy transfer (FRET)-based PA sensors. The former involves linking a PA-binding domain with a fluorescent protein, such as green fluorescent protein (Nakanishi et al., 2004; Corrotte et al., 2006; Du and Frohman, 2009; Bohdanowicz et al., 2013; Potocký et al., 2014; Li et al., 2023). FRET-based PA probes sandwich a PA-binding domain between two fluorophores, such as cyan and yellow fluorescent proteins, which serve as a donor and acceptor pair. The emission spectrum of the donor overlaps with the excitation spectrum of the acceptor (Li et al., 2019).

In addition, fluorescently labeled lipids, such as nitrobenzoxadiazole-PA and TopFluor tetramethylrhodamine-PA, have been used to study lipid distribution and dynamics in plants (Klymchenko and Kreder, 2014; Henkels et al., 2016; Kim et al., 2022). These labeled lipids can also be paired with cyan fluorescent protein-tagged proteins in FRET assays to detect lipid–protein interactions (Yao and Wang, 2023). Such techniques have been used to investigate the spatiotemporal distribution of PA across different cells, tissues, organs, and developmental stages. Moreover, recent developments in using click chemistry for controlling and detecting PA production offer promising tools for visualizing the cellular production, subcellular distribution, and cellular interactions of PA (Tei and Baskin, 2022).

PA MODULATION OF SEXUAL REPRODUCTION

Plant sexual reproduction begins with gametogenesis, followed by the pollen–stigma interaction, pollen germination, and tube elongation. The pollen tube transports sperm cells through the pistil and style to the ovule, where fertilization occurs. The fertilized ovule then undergoes embryogenesis, ultimately developing into a seed and/or fruit that contains the seed. PA and its metabolizing enzymes are involved in various steps of the reproductive process (Figure 2).

PA in gametogenesis and pollen function

In *Arabidopsis,* clade II DGK2 and DGK4 are highly expressed during anther development and homozygous *dgk2dgk4* double mutants are gametophyte lethal (Angkawijaya et al., 2020). Heterozygous *dgk2dgk4* mutants show reduced pollen germination rates and pollen tube elongation compared to the wild type (WT), which can be rescued by the addition of di18:1 PA or di18:2 PA. Translocation-based lipid probes revealed that PA accumulates at the subapical plasma membrane of the growing pollen tube, whereas DAG localizes primarily at the apex, and both DAG and PA colocalize at the dome of the apex (Figure 2) (Potocký et al., 2014). In tobacco (*Nicotiana tabacum [Nt]),* DGK5 is associated with the subapical plasma membrane of the pollen tube. Overexpression of functional *NtDGK5*, but not the inactive $NtDGK5_{G188A}$, results in wavy pollen tube growth with increased pectin deposition on the tube cell wall (Scholz et al., 2022). DGK4 is found in the cytosol at the apex of the pollen tube in both *Arabidopsis* and pear (*Pyrus bretschneideri* [*Pbr*]) (Vaz Dias et al., 2019; Kong et al., 2021). The *dgk4* mutants exhibit diminished pollen tube elongation rates and impaired membrane recycling (Figure 2) (Vaz Dias et al., 2019). In pear, *DGK4* knockdown alters vacuole morphology in the pollen tube and induces nuclear DNA fragmentation (Kong et al., 2021). These results indicate that DGKs and PA are integral for successful pollen tube germination and elongation.

In *Arabidopsis*, on the other hand, heterozygous *lppα2–lppε1* double mutants also show reduced pollen germination and pollen tube growth (Nguyen and Nakamura, 2023). Knockdown of *LPPα2* in the *lppε1* mutant results in shrunken pollen grains. LPPs catalyze the dephosphorylation of PA to DAG, the reverse reaction to DGKs (Figure 1). Exogenous DAG rescues these defects, suggesting that LPPs and DAG promote pollen tube germination and elongation (Nguyen and Nakamura, 2023). In pepper (*Capsicum annuum [Ca]),* silencing *CaPI-PLC5*, which hydrolyzes PIPs to DAG, reduces the number of pollen grains, causes wrinkled pollen grains, and lowers pollen germination rates (Ma et al., 2024). In contrast, PLDδ activity suppresses pollen tube elongation, as *pldδ* mutants exhibit longer pollen tubes than the WT. This phenotype can be rescued by complementation with active PLDδ, but not with the inactive $PLD\delta_{R622D}$ mutant, suggesting that PLDδ-generated PA suppresses pollen tube elongation (Jia et al., 2021).

These studies indicate that PA and its metabolizing enzymes are involved in pollen development and tube elongation, although the underlying mechanisms remain unclear. Both PLDδ and DGKs produce PA but have opposite effects on pollen tube elongation. Similarly, DGKs and LPPs, which catalyze opposite reactions in PA and DAG metabolism, both promote pollen germination and pollen tube elongation. Lipidomic analysis of germinating pollen over time showed that several PA species, such as 34:2, 36:4, and 32:0, increase during pollen germination, while others (36:2, 36:3, 36:6, and 34:1) increase during pollen tube elongation. Conversely, PA species 34:3 and 36:5 decrease during pollen tube elongation (Serrano et al., 2022). These results suggest that the function of PA is context-dependent, influenced by spatiotemporal patterns, the substrates used, the specific PA species produced, and interactions with other cellular components and lipids.

PA in self-incompatibility (SI)

SI is a mechanism by which flowering plants prevent self-pollination, promoting outcrossing and genetic diversity. During SI, the allele-specific recognition of pollen-encoded ligands—S-locus Cys-rich proteins or S-locus protein 11—by the S-locus receptor kinase leads to the phosphorylation of the E3 ubiquitin ligase Armadillo repeat-containing 1, which degrades compatibility factors and leads to pollen rejection (Scandola and Samuel, 2019). Knockdown of *PLDα1* in rapeseed (*Brassica*

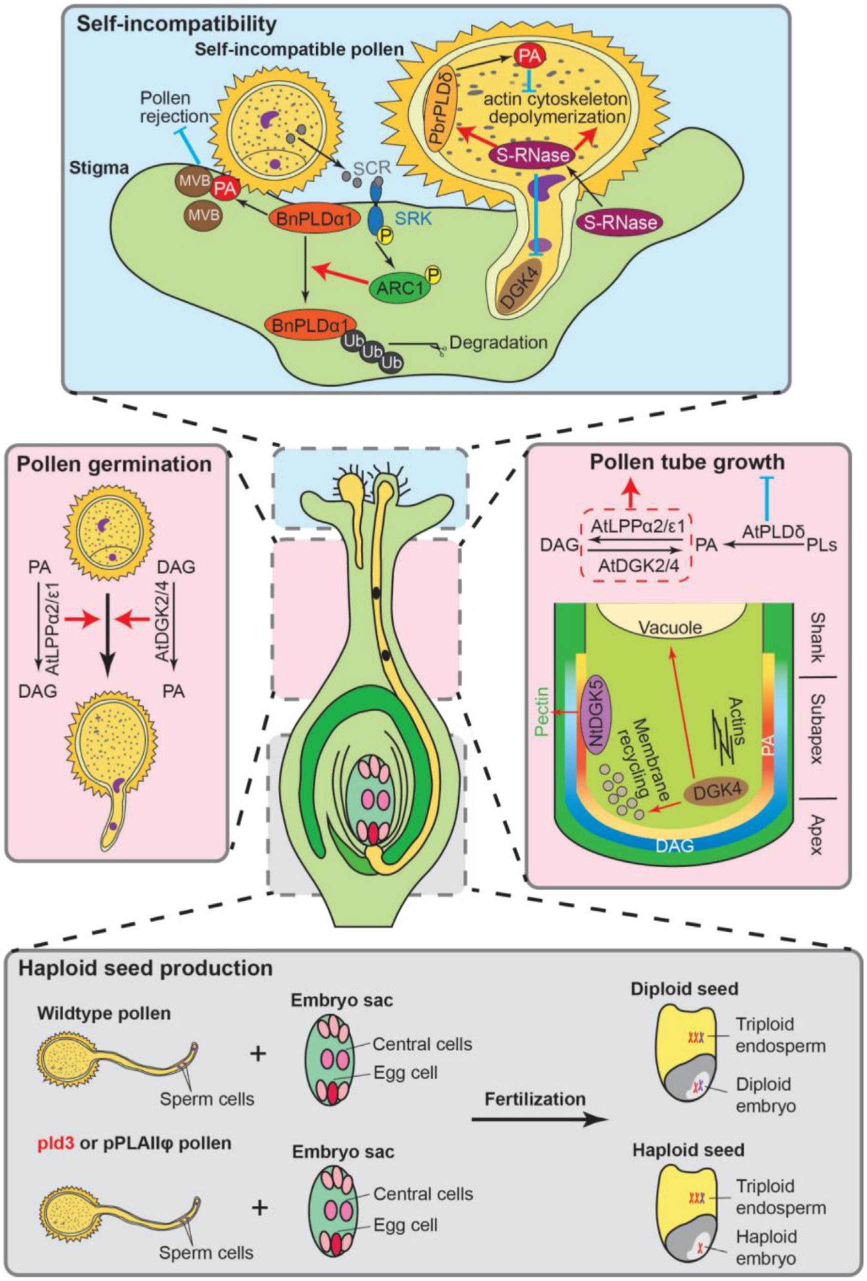

Figure 2. PA and its metabolizing enzymes at different steps of reproduction.
AtDGK2/4 and AtLPPα2/ε1, which catalyze opposite reactions in PA and DAG conversion, promote pollen germination and pollen tube elongation. The activity of AtPLDδ, which produces PA, suppresses pollen tube elongation. During pollen tube elongation, PA is more abundant at the subapical plasma membrane of the pollen tube, while DAG is mainly at the apex of the pollen tube. NtDGK5, associated with the subapical plasma membrane, increases pectin deposition in the pollen tube, while DGK4, localized to the cytosol at the apex, regulates vacuole morphology and membrane recycling. During the self-incompatibility response, the pollen-derived S-locus Cys-rich protein or S-locus protein 11 (SCR/SP11) is recognized by the S-locus receptor kinase (SRK), which leads to the phosphorylation of ARC1 (Armadillo repeat-containing 1). Phosphorylated ARC1 ubiquitinates and degrades PLDα1, inhibiting the exocytosis of multivesicular bodies and rejecting self-incompatible pollen. Conversely, the pistil-derived S-RNase interacts with actin, depolymerizing the cytoskeleton and triggering programmed cell death in incompatible pollen. S-RNase induces the expression of *PLDδ1* and increases PA production, which prevents the depolymerization of the actin cytoskeleton and delays SI signaling. S-RNase also reduces the abundance of PbrDGK4 at the cytosol of the pollen tube apex. The loss of pollen-specific *PLD3* or *pPLAIIϕ* in maize leads to maternal haploid seed formation. Red arrows and blue T-shaped lines indicate activation and inhibition of targets, respectively. At: *Arabidopsis thaliana*; Bn: *Brassica napus*; DGK: DAG kinase; LPP: lipid phosphate phosphatase; MVB: multivesicular body; Nt: *Nicotiana tabacum*; PA: phosphatidic acid; Pbr: *Pyrus bretschneideri*; pPLAIIϕ: patatin-related phospholipase AIIϕ; PLD: phospholipase D; Ub: ubiquitin.

napus [Bn]) decreases the number of compatible pollen grains adhering to the stigma and the length of pollen tube (Scandola and Samuel, 2019). The application of PA rescues these compatible pollination defects in *PLDα1*-knockdown lines. Overexpression (OE) of *PLDα1* and the exogenous application of PA increase the attachment of incompatible pollen to the stigma and promote pollen tube growth, thus breaking down the SI response. During the SI response, the ubiquitination of PLDα1 by Armadillo repeat-containing 1 leads to the degradation of PLDα1, which inhibits the exocytosis of multivesicular bodies and results in the rejection of self-incompatible pollen (Scandola and Samuel, 2019).

Furthermore, in rapeseed, treatment with NaCl, which disrupts the SI response, increases the expression of *PLDα1* and the production of PA, which binds to mitogen-activated protein kinase 6, leading to the breakdown of SI (Li et al., 2024c). Additionally, the female SI determinant S-RNase induces the expression of *PLDδ1*, leading to elevated PA production and stabilization of the actin cytoskeleton. This stabilization delays SI signaling and prevents actin depolymerization (Chen et al., 2018). S-RNase is secreted by the pistil and translocated into the growing pollen tube, where it interacts with actin to depolymerize the actin cytoskeleton and cause programmed cell death (Chen et al., 2018). In contrast, S-RNase reduces the abundance of PbrDGK4, another PA-producing enzyme, in the cytosol at the apex of the pollen tube (Kong et al., 2021).

These results indicate that PA and specific PLDs suppress SI, potentially through PA's stabilization of the actin cytoskeleton (Figure 2). While the actin-severing activity of S-RNase promotes SI by inhibiting pollen tube growth and inducing cell death in self-incompatible pollen, the activation of PLDs and elevated PA levels are associated with dampening SI (Chen

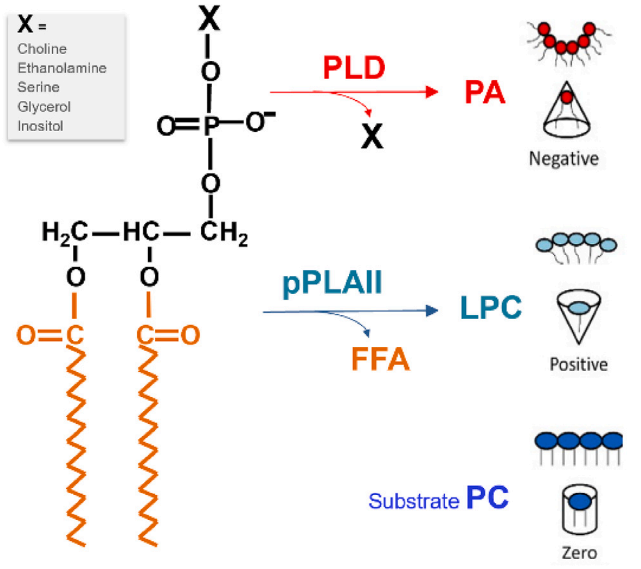

Figure 3. Hydrolysis of lipids by PLD and pPLAII and its impacts on membrane structure.
FFA: free fatty acid; pPLAII: patatin-related phospholipase AII; LPC: lysophosphatidylcholine; PLD: phospholipase D. The substrate phosphatidylcholine (PC) is used as an example.

et al., 2018). Other studies have shown that PA interacts with cytoskeletal proteins, such as capping proteins and MAP65-1, to regulate actin and microtubule organization and dynamics. PA inhibits the activities of capping proteins to promote actin polymerization (Huang et al., 2006) and binds to MAP65-1 to enhance microtubule polymerization and bundling (Zhang et al., 2012). In addition, certain PLDs have been found to associate with microtubules or actin filaments (Gardiner et al., 2001; Kusner et al., 2003; Song et al., 2020). Thus, the suppression of SI by PLDs and PA may involve their impact on cytoskeletal polymerization and stabilization during the SI response.

PLD in haploid seed production and embryogenesis

Sexual reproduction combines the genomes of two parents, driving evolution and plant breeding. Recent studies indicate that pollen-specific phospholipases are involved in paternal genome transmission, as their loss results in some embryos lacking the paternal genome (*i.e.*, maternal haploid embryoid seeds) (Figure 2) (Gilles et al., 2017; Kelliher et al., 2017; Liu et al., 2017; Li et al., 2021). This discovery emerged from the cloning of the mutant *MATRILINEAL* (*MATL*) gene, which has been used as a haploid inducer in maize breeding. *MATL* (also known as *NOT LIKE DAD*/*ZmPLA1*) encodes a pollen-specific patatin-related phospholipase A (pPLAIIϕ), which hydrolyzes phospholipids to lysophospholipids (LPLs) and free fatty acids (FFAs). Recently, similar pPLAIIϕ-mediated haploid induction has been demonstrated in other cereal crops, including rice and wheat (Yao et al., 2018; Liu et al., 2020).

The loss of *pPLAIIϕ* increases the expression of *PLD3, a* pollen-specific *PLD* (Kelliher et al., 2017). Knockout (KO) of *PLD3* in maize (*Zea mays* [*Zm*]) leads to haploid induction (Li et al., 2021). PLD3 and MATL synergistically enhance haploid induction rates (HIRs): single mutants of *zmmatl* and *zmpld3* show HIRs of 1.2% and 0.96%, respectively, whereas double mutants exhibit an HIR of about 4%. The DOMAIN OF UNKNOWN FUNCTION membrane protein (DMP) is also involved in haploid induction. *zmpld3–zmdmp* double mutants have similar HIRs to *zmpld3* single mutants, and the *zmpld3* (+/−)–*zmmatl–zmdmp* triple mutants exhibit an HIR of about 7% (Li et al., 2021). These findings indicate that these phospholipases are essential for paternal genome transmission during sexual reproduction.

The mechanisms by which the loss of *pPLAIIϕ* and *PLD* leads to maternal haploid seed formation are still unclear. pPLAIIϕ hydrolyzes common membrane phospholipids to produce LPLs and FFAs (Figure 3, middle), whereas PLD hydrolyzes phospholipids to produce PA (Figure 3, top). LPLs and PA significantly impact membrane shapes, which are important for vesicular trafficking, as well as membrane fusion and fission (McMahon and Boucrot, 2015; Zhukovsky et al., 2019; Kim and Wang, 2020). The substrate PC is cylindrical, forming a flat bilayer, whereas the product of pPLAIIϕ-catalyzed reaction, lysophosphatidylcholine (LPC), has a large headgroup relative to its acyl chain size. This size difference confers an inverted conical shape to LPC, inducing membranes to bend into positive curvature. Conversely, PA, the product of PLD3-catalyzed reaction, with its small headgroup-to-acyl-chain ratio, confers a conical shape, causing membranes to bend into negative curvature (Figure 3). This bending of membranes is crucial for bringing them together, facilitating sperm-egg cell fusion during fertilization; the greater the curvature of the membrane, the more fusogenic it becomes (Stein et al., 2004; Atif et al., 2006).

In addition, PA/LPC-mediated curvature could also affect the interaction between proteins and membranes. Both lipids and proteins may mediate close membrane apposition and induce membrane curvature to drive membrane fusion. The sperm-specific DMP is regarded as a helper in membrane fusion (Cyprys et al., 2019; Zhong et al., 2020). While the loss of *DMP* alone has minimal effects on haploid induction, combining the *pplaIIϕ* and *dmp* mutants significantly increases haploid induction rates, which is widely applied in maize haploid induction breeding (Jacquier et al., 2020). Lipidomic analysis of maize *pplaiiϕ mutant pollen has* revealed significantly decreased LPC and lysophosphatidylethanolamine levels and increased DAG levels (Jiang et al., 2022). Localization studies showed that pPLAIIϕ is localized at the peri-germ cell membrane of the pollen, surrounding the sperm cells, but not at the plasma membrane of sperm cells (Gilles et al., 2021; Sugi et al., 2024). Whether PLD3 is associated with sperm cells remains unknown; however, its effects on membrane composition may extend beyond its immediate localization due to the mobility of PA and LPLs (Wang et al., 2006; Kim and Wang, 2020; Noack and Jaillais, 2020). Thus, it remains to be determined whether the pollen-specific pPLAIIϕ and PLD3 affect the lipid composition and functions of sperm cell membranes.

Interestingly, some studies indicated that the *pPLAIIϕ* mutant leads to post-fertilization chromosome elimination (Zhao et al., 2013; Kelliher et al., 2019), while others reported continuous chromosome fragmentation occurrs post-meiosis (Li et al., 2017; Sun et al., 2022). These results suggest that

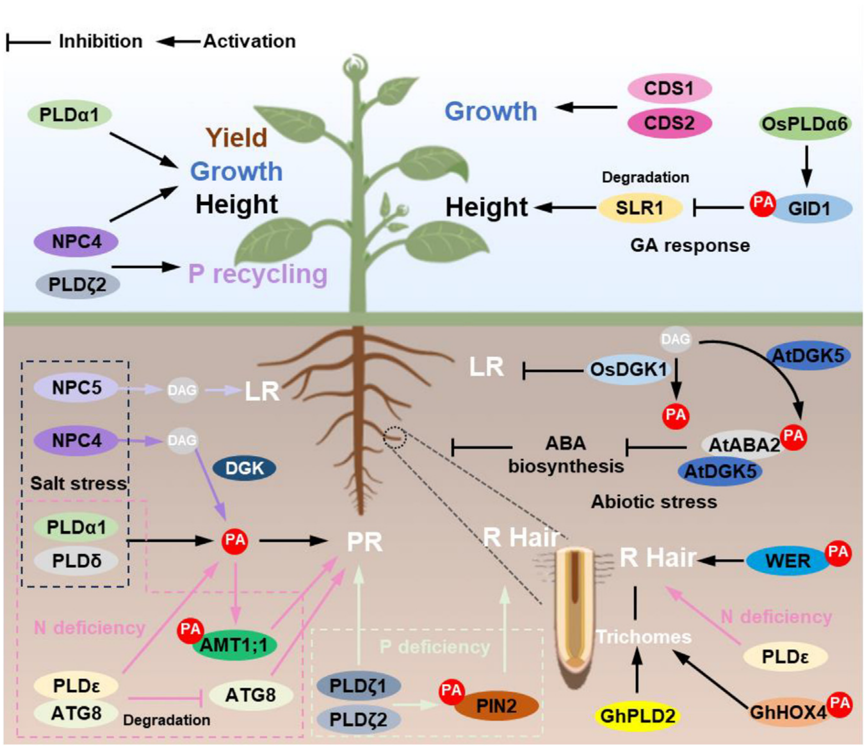

Figure 4. Impacts of PA and its metabolizing enzymes in root and above-ground architecture.
OsDGK1 KO blocks the conversion of DAG to PA and promotes the development of LR, while AtDGK5 interacts with ABA2 to regulate ABA biosynthesis, influencing root growth under abiotic stress. Under salt stress, NPC4 and NPC5 regulate the growth of PR and LR through PA and DAG, respectively. PA, PA target proteins, and PA metabolic enzymes also maintain the development of PR, LR, and root hairs in response to nutritional stress. PA produced by OsPLDα6 regulates the stability of SLR1 through its target protein GID1, affecting GA response and plant height. NPC4, PLDα1, and PLDζ2 positively affect plant growth, plant height, and yield. Arrows represent activation; T-shaped lines represent inhibition. ABA2: ABA-DEFICIENT 2; AMT1;1: ammonium transporter 1;1; At: *Arabidopsis thaliana*; CDS: cytidine diphosphate synthase; ATG8: autophagy-related protein 8; DAG: diacylglycerol; DGK: DAG kinase; GA: gibberellin; Gh: *Gossypium hirsutum*; GID1: GA-INSENSITIVE DWARF1; HOX4: a homeodomain-leucine zipper IV transcription factor; LR: lateral root; NPC: nonspecific phospholipase C; Os, *Oryza sativa;* PR: primary root; R: root. LPP: lipid phosphate phosphatase; MVB: multivesicular body; PA: phosphatidic acid; PLD: phospholipase D; SLR1: SLENDER RICE1; WER: WEREWOLF.

pPLAIIϕ maintains sperm genome stability, as DNA damage can occur before or after fertilization. Increased reactive oxygen species (ROS) levels are implicated in causing DNA damage, chromosome fragmentation, and subsequent genome elimination in the *mtl* mutant (Jiang et al., 2022; Sun et al., 2022). The loss of *pPLAIIϕ* increases *PLD3* expression, which may potentially enhance PA production. PA is known to increase ROS production by activating NADPH oxidases (Zhang et al., 2009; Kong et al., 2024). Elevated PA may increase ROS levels, leading to DNA damage and chromosomal damage and elimination. While this appears to contradict the synergistic effect of *pPLAIIϕ* and *PLD3* on haploid induction, PA is known to mediate both ROS production and cellular responses to ROS. For instance, the loss of *PLDδ* in *Arabidopsis* increases sensitivity to ROS-induced DNA damage and cell death (Zhang et al., 2003). Thus, the changes in PA, LPLs, and FFAs, due to the loss of *pPLAIIϕ* and *PLD3*, could have diverse effects before and after fertilization, though the mechanisms remain unresolved.

During embryogenesis, reduced expression of *CDS1* and *CDS2* in *cds1-cds2* double mutants, which convert PA to CDP-DAG for the biosynthesis of PG, PI, and PS (Figure 1), delays embryonic development in *Arabidopsis* (Du et al., 2022). Using microspore embryogenesis, where immature male gametophytes develop into embryos *in vitro*, this study detected changes in PA, but not in PC, PE, PG, or PS during the globular and heart-shaped embryo stages in rapeseed (Du et al., 2022). Further studies are needed to determine whether and how PA is directly involved in embryogenesis.

EFFECTS OF PA ON PLANT ARCHITECTURE

Plant growth patterns and morphological features are influenced by genetic and environmental factors. PA and its metabolizing enzymes modulate root and above-ground growth and architecture not only in response to stress conditions such as salinity, drought, and nutrient deficiency but also under optimal, unstressed conditions (Figure 4).

PA in root growth and architecture

Plant root systems play critical roles in anchorage, water and nutrient uptake, and responses to environmental changes. The analysis of DGKs in rice (*Oryza sativa* [*Os*]) revealed that OsDGK1 and its derived PA play an important role in root architecture under unstressed conditions (Yuan et al., 2019). *OsDGK1*-KO plants have more lateral roots (LRs) and a smaller radius of the primary root (PR; known as the seminal root in rice) than the WT, whereas the OE of *OsDGK1* results in fewer LRs with a larger PR radius. Supplying PA restores the root phenotype of *OsDGK1*-KO to that of the WT (Yuan et al., 2019). In contrast to the inhibitory effect of PA on LR development, PA promotes PR elongation. These results indicate that DGK and its product PA play important roles in regulating root architecture, but the mechanism remains unknown. A recent study showed that DGK5 in *Arabidopsis* affects root growth under stress but not under normal growth conditions (Li et al., 2024a). In the presence of NaCl or sorbitol, *DGK5*-KO plants have more and longer roots than the WT, whereas its OE shows the opposite phenotypes. These results suggest that DGK5

suppresses overall root growth in *Arabidopsis* in response to hyperosmotic stress. Further analyses showed that DGK5 and its lipid product PA suppress ABA production by interacting with ABA-DEFICIENT 2, a key ABA biosynthesis enzyme. Both external and internal cues, such as environmental stresses and hormones, impact root growth and patterning. ABA plays an important role in the plant response to stress, and increased ABA production likely contributes to the increased root growth observed in the *DGK5*-KO under stress (Li et al., 2024a).

In addition, several PLDs, which hydrolyze phospholipids to produce PA, are involved in stress-affected root growth. The *pldα1pldδ* mutants, which have lower PA contents, exhibit shorter PRs under salt stress. Exogenous PA restores the PR length of *pldα1pldδ* to WT levels under salt stress (Wang et al., 2019). Moreover, PLDα- and PLDδ-derived PA interacts with ammonium transporter 1;1 to mediate NH_4^+ uptake in *Arabidopsis* (Wang et al., 2019; Cao et al., 2023). The loss of *PLDζ1* and *-2* in *Arabidopsis* decreases the PR length under phosphate deficiency (Li et al., 2006a, 2006b; Su et al., 2018). Conversely, OE of *PLDε*, which increases PA levels, increases PR length in *Arabidopsis*, canola, and soybean (Hong et al., 2009; Lu et al., 2016; Yao et al., 2022a, 2022b).

Furthermore, specific NPCs affect stress-impacted root growth and patterning. NPC4 is involved in the plant's response to phosphorus efficiency and salinity. Earlier studies showed that the KO of *NPC4* decreases PR growth under salt stress in *Arabidopsis*. Supplying PA and DAG, but not DAG plus a DGK inhibitor, restores the PR growth of the *NPC4*-KO under salt stress, suggesting that NPC4 functions through DGK-mediated PA production from DAG (Peters et al., 2010). Conversely, NPC5 and its derived DAG promote LR formation and growth under salt stress (Peters et al., 2014). These results suggest that PA and DAG have distinct functions in LR and PR growth.

These studies revealed that the interactions of PA and its metabolizing enzymes with proteins involved in hormone action and production form the basis for the effects of PA on stress-affected growth and morphological responses. ABA and auxin play important, often antagonistic roles in modulating root architecture. For example, in *Arabidopsis*, auxin promotes LR initiation and affects PR and LR growth (Liu and von Wirén, 2022), whereas ABA inhibits LR and PR growth, with LRs being more sensitive to ABA than PRs (Thole et al., 2014; Waidmann et al., 2020; Qin et al., 2023). ABA biosynthesis and signaling pathways are activated under various stress conditions, affecting root growth and patterning (Harris, 2015). PA and DGK5 bind to ABA-DEFICIENT 2 and suppress ABA production (Li et al., 2024a), whereas PA binds PINOID kinase and promotes the PINOID kinase-dependent phosphorylation of PIN, which regulates auxin efflux and redistribution (Wang et al., 2019). In addition, NPC3 and NPC4 interact with PIN-FORMED2 to promote auxin transport and signaling (Li et al., 2024b). Furthermore, PLDα6-derived PA in rice binds to the gibberellin (GA) receptor GA-INSENSITIVE DWARF1 (GID1) and mediates GA signaling to enhance plant height and the longitudinal growth of reproductive structures (Cao et al., 2021; Figure 4).

PA in plant height and reproductive architecture

Several PA-producing enzymes have been reported to affect above-ground plant growth and patterning. Rapeseed, a tetraploid species, contains 32 *PLD* genes. The KO of four *PLDα1* genes via CRISPR–Cas9-mediated genome editing results in shorter aerial organs, as well as reduced plant height, inflorescence length, number of effective (seed-bearing) branches, silique length, and seed yield per plant (Lu et al., 2019). These results indicate that an appropriate dosage of PLDα1 is necessary to maintain normal growth in rapeseed. A subsequent study with rice *PLD* mutants showed that PLDα6 influences longitudinal growth and development by modulating GA signaling through PA (Cao et al., 2021). GAs are essential hormones that regulate plant growth and development throughout the life cycle (Hu et al., 2018). *PLDα6*-KO plants exhibit reduced sensitivity to GA compared to the WT, and exogenous PA restores the mutant's normal GA response. At maturity, longitudinal growth—including plant height, panicle length, and flag leaf length—is significantly reduced in the *pldα6 mutant compared* to the WT. Further analyses indicated that PLDα6 and PA regulate the distribution of the soluble GA receptor GID1. PA binds to GID1, which is essential for GID1's nuclear localization, and the loss of *PLDα6* impairs GA-induced nuclear localization of GID1. In addition, *PLDα6*-KO plants exhibit attenuated GA-induced degradation of the DELLA protein SLENDER RICE1. These data suggest that PLDα6 and PA positively mediate GA signaling to enhance longitudinal growth and development (Figure 4) (Cao et al., 2021).

In addition, BnaNPC4 promotes growth and seed production in rapeseed (Yang et al., 2023). The hypocotyl length is longer in *BnaNPC4*-OE lines but shorter in KO lines compared to the WT. *BnaNPC4*-OE lines display significant improvements in growth, develop more effective branches (seed-bearing branches) and siliques, and show increased seed yield per plant, whereas *BnaNPC4*-KO lines exhibit the opposite traits under the same conditions (Yang et al., 2023). Similarly, the OE of *NPC4* promotes growth and development in camelina (Li et al., 2025). *NPC4* OE lines exhibit increased DAG levels and decreased PA/DAG ratios in both old and young leaves compared to those of the WT. Additionally, they flower approximately two days earlier, have more branches, and exhibit increased seed yield per plant, oil content, and oil yield per plant. Moreover, the effect of NPC4 is more pronounced under phosphorus-deficient than -sufficient conditions, highlighting its positive role in the plant's response to low phosphorus availability (Li et al., 2025). Furthermore, PLDζ2- and NPC4-mediated hydrolysis of membrane phospholipids promotes the remobilization of phosphorus from senescent leaves to growing tissues, indicating that phospholipid hydrolysis-mediated phosphorus recycling enhances phosphorus use efficiency in plants (Yang et al., 2024).

PA in root hairs and trichomes

Root hairs are specialized, single-celled outgrowths from the epidermis of a plant root, primarily responsible for absorbing water and nutrients from the soil (Karlova et al., 2021). An earlier study showed that PA interacts with WEREWOLF, a Myb transcription factor (TF), to regulate root hair patterning (Yao et al., 2013). Comparative lipidomic profiling of soybean root hairs versus roots stripped of root hairs revealed that the membrane glycerolipidomes, particularly PA, in root hairs are

more responsive to variations in nitrogen (N) and phosphate (P_i) availability than those in the rest of the root system (Wei et al., 2016). Another study indicated that PLDζ-derived PA affects the vacuolar degradation of the auxin efflux carrier PIN-FORMED2, thereby promoting root hair development in *Arabidopsis* under low-phosphorus conditions (Figure 4) (Lin et al., 2020).

Trichomes are epidermal, hair-like structures similar to root hairs, but they occur on the aerial parts of plants, such as leaves and stems. They often serve functions such as defense and reducing water loss through transpiration (Han et al., 2022). Genetic analysis of *Arabidopsis* mutants suggested that similar molecular mechanisms mediate the differentiation of trichomes and root hair cells. PLD and PA are also involved in the development of cotton (*Gossypium hirsutum [Gh]*) fibers, which are unicellular and highly elongated trichomes on seeds (Wang et al., 2024b). Previous studies on multiple stable quantitative trait loci have revealed the role of *PLD* genes in fiber development. Virus-induced gene silencing of *GhPLD2* in cotton seedlings results in reduced ROS levels, impaired anther dehiscence, and disrupted fiber initiation and elongation (Ma et al., 2021). PA interacts with the homeodomain-leucine zipper IV TF GhHOX4, which is known to promote fiber elongation. This interaction suppresses the transcriptional regulation of GhHOX4 on downstream genes during the transition from fiber elongation to secondary cell wall thickening in fiber development (Wang et al., 2024b).

These findings, along with other studies, revealed that PA signaling operates within nucleiopening new directions for investigating the regulatory functions of PA (Yao et al., 2024). Nuclear PA can be produced extranuclearly or by enzymes associated with nuclei. PA is highly mobile, capable of traversing membranes and promoting vesicle budding and fusion. Under heat stress, PA produced by PLDδ at the plasma membrane rapidly translocates to the nucleus, a process required for the heat-induced nuclear translocation of cytosolic glyceraldehyde-3-phosphate dehydrogenase (GAPC) (Kim et al., 2022; Li et al., 2023). PA has been shown to affect gene expression by directly binding to TFs or interacting with TF-associated proteins (Kim et al., 2019, 2022, 2023; Cai et al., 2020a). For example, PA interacts with and inhibits the binding of late elongated hypocotyl and circadian clock-associated 1, the key clock effectors that regulate hypocotyl elongation and plant height (Kim et al., 2019), to the promoters of their target genes, such as TIMING OF CAB EXPRESSION 1 and 3-ketoacyl-ACP synthase III (Kim et al., 2023), limiting their transcriptional activation. PA also interacts with AT-hook motif-containing nuclear localized 4, which suppresses lipid degradation and root growth during seed germination (Cai et al., 2020a). Furthermore, PA binds to GAPC to facilitate its nuclear translocation. Once in the nucleus, GAPC interacts with nuclear factor Y subunit C10 to mediate the plant's response to high temperatures (Kim et al., 2020, 2022). These nuclear functions of PA highlight its role in regulating gene expression, cell proliferation, and stress responses.

PERSPECTIVES

Recent results indicate that PA plays important roles in plant architecture and sexual reproduction, directly influencing seed production. PA and its metabolizing enzymes are involved in multiple reproductive steps, including gametogenesis, pollen tube growth, SI, haploid embryo formation, embryogenesis, and seed development. They also influence plant reproductive and root architecture. However, the understanding of PA's role in reproduction and architecture is still incomplete and fragmented, and many questions remain to be addressed, particularly regarding the detailed mechanisms by which PA impacts these processes.

The mode of PA's action is multifaceted. Metabolically, PA is a central metabolite in lipid metabolism, and some of its effects may result from its role in the biosynthesis and homeostasis of membrane and storage lipids. At the membrane and subcellular levels, PA accumulation affects membrane curvature, vesicular trafficking, cytoskeletal dynamics, and protein interactions with membranes. At the molecular level, PA directly binds to target proteins, affecting protein–DNA interactions and gene expression. The phosphate head group of PA undergoes pH-dependent dual deprotonation, allowing it to switch between electrostatic and hydrogen-bonding interactions with effector proteins (Kooijman et al., 2007). These biophysical properties distinguish PA from other membrane phospholipids in protein interactions. The PA–protein interactions can modulate a protein's enzymatic activity, structure, membrane association, and/or interaction with other cellular components such as nucleic acids and chromatin. The multifaceted nature of PA presents challenges in pinpointing its precise molecular mechanisms. However, increasing evidence indicates that PA's mode of action in specific plant processes depends on where, when, and which PA species are produced in the cell, as well as the proteins they interact with.

Genetic manipulations of genes involved in PA metabolism have provided valuable insights into PA's involvement in specific plant processes. The application of multiplexed genome editing to modify various combinations of PA-metabolizing genes will be a powerful tool for uncovering PA's effects on specific processes (Kim et al., 2024). Advancements in the effective quantitative profiling of PA species and PA biosensors, along with developments in click chemistry for cell-type-specific detection of PA, will help reveal the dynamic changes in PA at both subcellular and submembrane levels. Integrating genetic, cell biological, and lipidomic approaches will provide further information about the mechanisms by which PA influences plant reproduction and architecture. In addition, PA's interaction with proteins has emerged as a key mechanism underlying its regulatory functions. Analyses of these lipid–protein interactions have begun to reveal how PA acts as a cellular mediator and affects plant reproduction and architecture. Moreover, PA-binding proteins exhibit varied binding preferences for different PA species, which could potentially explain the specificity and diverse functions of PA (Yao et al., 2024). Further exploration of PA interactomes and the production of specific PA species and their protein-binding partners would provide valuable insights into PA's functions. Furthermore, DAG is both a substrate and a product of PA metabolism (Figure 1) and functions as a cellular mediator. Alterations in PA-metabolizing enzymes affect PA/DAG homeostasis in the cell (Li et al., 2024a). However, the role of DAG as a mediator and the effect of PA/DAG homeostasis on these processes remain largely unknown in plants. Further studies on PA's

function will not only improve our understanding of the regulatory processes in plant sexual reproduction but also offer potential applications for improving crop seed production under challenging environmental conditions.

FUNDING

The research in X.W.'s lab was supported by grants from the National Institute of General Medical Sciences of the National Institutes of Health under award no. R01GM141374, the National Science Foundation under grant nos. 2222157 and 2302424, and the USDA National Institute of Food and Agriculture 2020-67013-30908/project accession no. 1022148.

ACKNOWLEDGMENTS

No conflict of interest declared.

AUTHOR CONTRIBUTIONS

S.Y., B.Y., and X.W. created the graphs and wrote the manuscript. J.L., Shan Tang, Shaohua Tang, and S.-C.K. contributed to the discussion, literature searches, and revision of the manuscript.

SUPPLEMENTAL INFORMATION

Supplemental information is available at *Plant Communications Online*.

Received: September 30, 2024
Revised: November 27, 2024
Accepted: December 23, 2024
Published: December 24, 2024

REFERENCES

Amokrane, L., Pokotylo, I., Acket, S., Ducloy, A., Troncoso-Ponce, A., Cacas, J.-L., and Ruelland, E. (2024). Phospholipid Signaling in Crop Plants: A Field to Explore. Plants **13**:1532. https://doi.org/10.3390/plants13111532.

Angkawijaya, A.E., Nguyen, V.C., Gunawan, F., and Nakamura, Y. (2020). A pair of Arabidopsis diacylglycerol kinases essential for gametogenesis and endoplasmic reticulum phospholipid metabolism in leaves and flowers. Plant Cell **32**:2602–2620. https://doi.org/10.1105/tpc.20.00251.

Atif, S.M., Hasan, I., Ahmad, N., Khan, U., and Owais, M. (2006). Fusogenic potential of sperm membrane lipids: Nature's wisdom to accomplish targeted gene delivery. FEBS Lett. **580**:2183–2190. https://doi.org/10.1016/j.febslet.2006.03.015.

Bohdanowicz, M., Schlam, D., Hermansson, M., Rizzuti, D., Fairn, G.D., Ueyama, T., Somerharju, P., Du, G., and Grinstein, S. (2013). Phosphatidic acid is required for the constitutive ruffling and macropinocytosis of phagocytes. Mol. Biol. Cell **24**:1700–S7. https://doi.org/10.1091/mbc.e12-11-0789.

Cai, G., Kim, S.C., Li, J., Zhou, Y., and Wang, X. (2020a). Transcriptional regulation of lipid catabolism during seedling establishment. Mol. Plant **13**:984–1000. https://doi.org/10.1016/j.molp.2020.04.007.

Cai, G., Fan, C., Liu, S., Yang, Q., Liu, D., Wu, J., Li, J., Zhou, Y., Guo, L., and Wang, X. (2020b). Nonspecific phospholipase C6 increases seed oil production in oilseed Brassicaceae plants. New Phytol. **226**:1055–1073. https://doi.org/10.1111/nph.16473.

Cao, H., Liu, Q., Liu, X., Ma, Z., Zhang, J., Li, X., Shen, L., Yuan, J., and Zhang, Q. (2023). Phosphatidic acid regulates ammonium uptake by interacting with AMMONIUM TRANSPORTER 1; 1 in Arabidopsis. Plant Physiol. **193**:1954–1969. https://doi.org/10.1093/plphys/kiad421.

Cao, H., Gong, R., Yuan, S., Su, Y., Lv, W., Zhou, Y., Zhang, Q., Deng, X., Tong, P., Liang, S., et al. (2021). Phospholipase Dα6 and phosphatidic acid regulate gibberellin signaling in rice. EMBO Rep. **22**:e51871. https://doi.org/10.15252/embr.202051871.

Chen, J., Wang, P., de Graaf, B.H.J., Zhang, H., Jiao, H., Tang, C., Zhang, S., and Wu, J. (2018). Phosphatidic acid counteracts S-RNase signaling in pollen by stabilizing the actin cytoskeleton. Plant Cell **30**:1023–1039. https://doi.org/10.1105/tpc.18.00021.

Corrotte, M., Chasserot-Golaz, S., Huang, P., Du, G., Ktistakis, N.T., Frohman, M.A., Vitale, N., Bader, M.F., and Grant, N.J. (2006). Dynamics and function of phospholipase D and phosphatidic acid during phagocytosis. Traffic **7**:365–377. https://doi.org/10.1111/j.1600-0854.2006.00389.x.

Craddock, C.P., Adams, N., Bryant, F.M., Kurup, S., and Eastmond, P.J. (2015). Regulation of endomembrane biogenesis in arabidopsis by phospatidic acid hydrolase. Plant Signal. Behav. **10**:e1065367. https://doi.org/10.1080/15592324.2015.1065367.

Cyprys, P., Lindemeier, M., and Sprunck, S. (2019). Gamete fusion is facilitated by two sperm cell-expressed DUF679 membrane proteins. Nat. Plants **5**:253–257. https://doi.org/10.1038/s41477-019-0382-3.

Du, G., and Frohman, M.A. (2009). A lipid-signaled myosin phosphatase surge disperses cortical contractile force early in cell spreading. Mol. Biol. Cell **20**:200–208. https://doi.org/10.1091/mbc.e08-06-0555.

Du, X.Q., Yao, H.Y., Luo, P., Tang, X.C., and Xue, H.W. (2022). Cytidinediphosphate diacylglycerol synthase—Mediated phosphatidic acid metabolism is crucial for early embryonic development of Arabidopsis. PLoS Genet. **18**:e1010320. https://doi.org/10.1371/journal.pgen.1010320.

Eastmond, P.J., Quettier, A.-L., Kroon, J.T.M., Craddock, C., Adams, N., and Slabas, A.R. (2010). PHOSPHATIDIC ACID PHOSPHOHYDROLASE1 and 2 regulate phospholipid synthesis at the endoplasmic reticulum in Arabidopsis. Plant Cell **22**:2796–2811. https://doi.org/10.1105/tpc.109.071423.

Gardiner, J.C., Harper, J.D., Weerakoon, N.D., Collings, D.A., Ritchie, S., Gilroy, S., Cyr, R.J., and Marc, J. (2001). A 90-kD Phospholipase D from Tobacco Binds to Microtubules and the Plasma Membrane. Plant Cell **13**:2143–2158. https://doi.org/10.1105/tpc.010114.

Ge, H., Chen, C., Jing, W., Zhang, Q., Wang, H., Wang, R., and Zhang, W. (2012). The rice diacylglycerol kinase family: functional analysis using transient RNA interference. Front. Plant Sci. **3**:60. https://doi.org/10.3389/fpls.2012.00060.

Gilles, L.M., Calhau, A.R.M., La Padula, V., Jacquier, N.M.A., Lionnet, C., Martinant, J.P., Rogowsky, P.M., and Widiez, T. (2021). Lipid anchoring and electrostatic interactions target NOT-LIKE-DAD to pollen endo-plasma membrane. J. Cell Biol. **220**:e202010077. https://doi.org/10.1083/jcb.202010077.

Gilles, L.M., Khaled, A., Laffaire, J.B., Chaignon, S., Gendrot, G., Laplaige, J., Bergès, H., Beydon, G., Bayle, V., Barret, P., et al. (2017). Loss of pollen-specific phospholipase NOT LIKE DAD triggers gynogenesis in maize. EMBO J. **36**:707–717. https://doi.org/10.15252/embj.201796603.

Gong, Q., Yao, S., Wang, X., and Li, G. (2024). Fine-tuning phosphatidic acid production for optimal plant stress responses. Trends Biochem. Sci. **49**:663–666. https://doi.org/10.1016/j.tibs.2024.05.008.

Han, G., Li, Y., Yang, Z., Wang, C., Zhang, Y., and Wang, B. (2022). Molecular Mechanisms of Plant Trichome Development. Front. Plant Sci. **13**:910228. https://doi.org/10.3389/fpls.2022.910228.

Harris, J.M. (2015). Abscisic acid: hidden architect of root system structure. Plants **4**:548–572. https://doi.org/10.3390/plants4030548.

Henkels, K.M., Miller, T.E., Ganesan, R., Wilkins, B.A., Fite, K., and Gomez-Cambronero, J. (2016). A Phosphatidic Acid (PA) conveyor system of continuous intracellular transport from cell membrane to nucleus maintains EGF receptor homeostasis. Oncotarget **7**:47002–47017. https://doi.org/10.18632/oncotarget.9685.

Hong, Y., Yuan, S., Sun, L., Wang, X., and Hong, Y. (2018). Cytidinediphosphate-diacylglycerol synthase 5 is required for

phospholipid homeostasis and is negatively involved in hyperosmotic stress tolerance. Plant J. **94**:1038–1050. https://doi.org/10.1111/tpj.13916.

Hong, Y., Devaiah, S.P., Bahn, S.C., Thamasandra, B.N., Li, M., Welti, R., and Wang, X. (2009). Phospholipase Dε and phosphatidic acid enhance Arabidopsis nitrogen signaling and growth. Plant J. **58**:376–387. https://doi.org/10.1111/j.1365-313x.2009.03788.x.

Hu, Y., Zhou, L., Huang, M., He, X., Yang, Y., Liu, X., Li, Y., and Hou, X. (2018). Gibberellins play an essential role in late embryogenesis of Arabidopsis. Nat. Plants **4**:289–298. https://doi.org/10.1038/s41477-018-0143-8.

Huang, S., Gao, L., Blanchoin, L., and Staiger, C.J. (2006). Heterodimeric capping protein from Arabidopsis is regulated by phosphatidic acid. Mol. Biol. Cell **17**:1946–1958. https://doi.org/10.1091/mbc.e05-09-0840.

Jacquier, N.M.A., Gilles, L.M., Pyott, D.E., Martinant, J.-P., Rogowsky, P.M., and Widiez, T. (2020). Puzzling out plant reproduction by haploid induction for innovations in plant breeding. Nat. Plants **6**:610–619. https://doi.org/10.1038/s41477-020-0664-9.

Jeannette, E., Paradis, S., and Zalejski, C. (2010). Diacylglycerol Pyrophosphate, A Novel Plant Signaling Lipid. Lipid Signaling in Plants, 263–276. https://doi.org/10.1007/978-3-642-03873-0_17.

Jia, Q., Zhang, S., Lin, Y., Zhang, J., Li, L., Chen, H., and Zhang, Q. (2021). Phospholipase Dδ regulates pollen tube growth by modulating actin cytoskeleton organization in Arabidopsis. Plant Signal. Behav. **16**:1915610. https://doi.org/10.1080/15592324.2021.1915610.

Jiang, C., Sun, J., Li, R., Yan, S., Chen, W., Guo, L., Qin, G., Wang, P., Luo, C., Huang, W., et al. (2022). A reactive oxygen species burst causes haploid induction in maize. Mol. Plant **15**:943–955. https://doi.org/10.1016/j.molp.2022.04.001.

Karlova, R., Boer, D., Hayes, S., and Testerink, C. (2021). Root plasticity under abiotic stress. Plant Physiol. **187**:1057–1070. https://doi.org/10.1093/plphys/kiab392.

Kelliher, T., Starr, D., Richbourg, L., Chintamanani, S., Delzer, B., Nuccio, M.L., Green, J., Chen, Z., McCuiston, J., Wang, W., et al. (2017). MATRILINEAL, a sperm-specific phospholipase, triggers maize haploid induction. Nature **542**:105–109. https://doi.org/10.1038/nature20827.

Kelliher, T., Starr, D., Su, X., Tang, G., Chen, Z., Carter, J., Wittich, P.E., Dong, S., Green, J., Burch, E., et al. (2019). One-step genome editing of elite crop germplasm during haploid induction. Nat. Biotechnol. **37**:287–292. https://doi.org/10.1038/s41587-019-0038-x.

Kim, S.C., and Wang, X. (2020). Phosphatidic acid: an emerging versatile class of cellular mediators. Essays Biochem. **64**:533–546. https://doi.org/10.1042/ebc20190089.

Kim, S.C., Guo, L., and Wang, X. (2020). Nuclear moonlighting of cytosolic glyceraldehyde-3-phosphate dehydrogenase regulates Arabidopsis response to heat stress. Nat. Commun. **11**:3439. https://doi.org/10.1038/s41467-020-17311-4.

Kim, S.C., Nusinow, D.A., and Wang, X. (2024). Identification of Arabidopsis phospholipase Ds involved in circadian clock alterations using CRISPR/Cas9-based multiplex editing. Preprint at bioRxiv. https://doi.org/10.1101/2024.01.09.574824.

Kim, S.C., Yao, S., Zhang, Q., and Wang, X. (2022). Phospholipase Dδ and phosphatidic acid mediate heat-induced nuclear localization of glyceraldehyde-3-phosphate dehydrogenase in Arabidopsis. Plant J. **112**:786–799. https://doi.org/10.1111/tpj.15981.

Kim, S.C., Edgeworth, K.N., Nusinow, D.A., and Wang, X. (2023). Circadian clock factors regulate the first condensation reaction of fatty acid synthesis in Arabidopsis. Cell Rep. **42**:113483. https://doi.org/10.1016/j.celrep.2023.113483.

Kim, S.C., Nusinow, D.A., Sorkin, M.L., Pruneda-Paz, J., and Wang, X. (2019). Interaction and regulation between lipid mediator phosphatidic acid and circadian clock regulators. Plant Cell **31**:399–416. https://doi.org/10.1105/tpc.18.00675.

Klymchenko, A.S., and Kreder, R. (2014). Fluorescent probes for lipid rafts: from model membranes to living cells. Chem. Biol. **21**:97–113. https://doi.org/10.1016/j.chembiol.2013.11.009.

Kong, L., Ma, X., Zhang, C., Kim, S.I., Li, B., Xie, Y., Yeo, I.C., Thapa, H., Chen, S., Devarenne, T.P., et al. (2024). Dual phosphorylation of DGK5-mediated PA burst regulates ROS in plant immunity. Cell **187**:609–623.e21. https://doi.org/10.1016/j.cell.2023.12.030.

Kong, X.X., Mei, J.W., Zhang, J., Liu, X., Wu, J.Y., and Wang, C.L. (2021). Turnover of diacylglycerol kinase 4 by cytoplasmic acidification induces vacuole morphological change and nuclear DNA degradation in the early stage of pear self-incompatibility response. J. Integr. Plant Biol. **63**:2123–2135. https://doi.org/10.1111/jipb.13180.

Kooijman, E.E., Tieleman, D.P., Testerink, C., Munnik, T., Rijkers, D.T.S., Burger, K.N.J., and de Kruijff, B. (2007). An electrostatic/hydrogen bond switch as the basis for the specific interaction of phosphatidic acid with proteins. J. Biol. Chem. **282**:11356–11364. https://doi.org/10.1074/jbc.m609737200.

Kusner, D.J., Barton, J.A., Qin, C., Wang, X., and Iyer, S.S. (2003). Evolutionary conservation of physical and functional interactions between phospholipase D and actin. Arch. Biochem. Biophys. **412**:231–241. https://doi.org/10.1016/S0003-9861(03)00052-3.

Li, J., Yao, S., Kim, S.C., and Wang, X. (2024a). Lipid phosphorylation by a diacylglycerol kinase suppresses ABA biosynthesis to regulate plant stress responses. Mol. Plant **17**:342–358. https://doi.org/10.1016/j.molp.2024.01.003.

Li, J., Yao, S., Jonas, M., Kim, S.C., and Wang, X. (2025). Non-specific Phospholipase C4 Improves Phosphorus Remobilization From Old to Young Leaves in Camelina. Plant Cell Environ. **48**:297–309. https://doi.org/10.1111/pce.15122.

Li, M., Welti, R., and Wang, X. (2006a). Quantitative profiling of Arabidopsis polar glycerolipids in response to phosphorus starvation. Roles of phospholipases D ζ1 and D ζ2 in phosphatidylcholine hydrolysis and digalactosyldiacylglycerol accumulation in phosphorus-starved plants. Plant Physiol. **142**:750–761. https://doi.org/10.1104/pp.106.085647.

Li, M., Qin, C., Welti, R., and Wang, X. (2006b). Double knockouts of phospholipases D ζ 1 and D ζ 2 in Arabidopsis affect root elongation during phosphate-limited growth but do not affect root hair patterning. Plant Physiol. **140**:761–770. https://doi.org/10.1104/pp.105.070995.

Li, T., Xiao, X., Liu, Q., Li, W., Li, L., Zhang, W., Munnik, T., Wang, X., and Zhang, Q. (2023). Dynamic responses of PA to environmental stimuli imaged by a genetically encoded mobilizable fluorescent sensor. Plant Commun. **4**:100500. https://doi.org/10.1016/j.xplc.2022.100500.

Li, T., Zhang, S., Yao, S., Li, X., Jia, Q., Yuan, J., Zhang, W., Wang, X., and Zhang, Q. (2024b). Nonspecific phospholipases C3 and C4 interact with PIN-FORMED2 to regulate growth and tropic responses in Arabidopsis. Plant Cell **36**:2310–2327. https://doi.org/10.1093/plcell/koae071.

Li, W., Song, T., Wallrad, L., Kudla, J., Wang, X., and Zhang, W. (2019). Tissue-specific accumulation of pH-sensing phosphatidic acid determines plant stress tolerance. Nat. Plants **5**:1012–1021. https://doi.org/10.1038/s41477-019-0497-6.

Li, X., Meng, D., Chen, S., Luo, H., Zhang, Q., Jin, W., and Yan, J. (2017). Single nucleus sequencing reveals spermatid chromosome fragmentation as a possible cause of maize haploid induction. Nat. Commun. **8**:991. https://doi.org/10.1038/s41467-017-00969-8.

Li, Y., Zhang, W., Yang, Y., Liang, X., Lu, S., Ma, C., and Dai, C. (2024c). BnaPLDα1-BnaMPK6 Involved in NaCl-Mediated Overcoming of Self-Incompatibility in Brassica napus L. Plant Sci. **345**:112116. https://doi.org/10.1016/j.plantsci.2024.112116.

Li, Y., Lin, Z., Yue, Y., Zhao, H., Fei, X., E, L., Liu, C., Chen, S., Lai, J., and Song, W. (2021). Loss-of-function alleles of ZmPLD3 cause haploid induction in maize. Nat. Plants **7**:1579–1588. https://doi.org/10.1038/s41477-021-01037-2.

Liang, Y., Huang, Y., Liu, C., Chen, K., and Li, M. (2023). Functions and interaction of plant lipid signalling under abiotic stresses. Plant Biol. **25**:361–378. https://doi.org/10.1111/plb.13507.

Lin, D.L., Yao, H.Y., Jia, L.H., Tan, J.F., Xu, Z.H., Zheng, W.M., and Xue, H.W. (2020). Phospholipase D-derived phosphatidic acid promotes root hair development under phosphorus deficiency by suppressing vacuolar degradation of PIN-FORMED2. New Phytol. **226**:142–155. https://doi.org/10.1111/nph.16330.

Liu, C., Li, X., Meng, D., Zhong, Y., Chen, C., Dong, X., Xu, X., Chen, B., Li, W., Li, L., et al. (2017). A 4-bp insertion at ZmPLA1 encoding a putative phospholipase A generates haploid induction in maize. Mol. Plant **10**:520–522. https://doi.org/10.1016/j.molp.2017.01.011.

Liu, H., Wang, K., Jia, Z., Gong, Q., Lin, Z., Du, L., Pei, X., and Ye, X. (2020). Efficient induction of haploid plants in wheat by editing of TaMTL using an optimized Agrobacterium-mediated CRISPR system. J. Exp. Bot. **71**:1337–1349. https://doi.org/10.1093/jxb/erz529.

Liu, Y., and von Wirén, N. (2022). Integration of nutrient and water availabilities via auxin into the root developmental program. Curr. Opin. Plant Biol. **65**:102117. https://doi.org/10.1016/j.pbi.2021.102117.

Lu, S., Yao, S., Wang, G., Guo, L., Zhou, Y., Hong, Y., and Wang, X. (2016). Phospholipase Dε enhances Braasca napus growth and seed production in response to nitrogen availability. Plant Biotechnol. J. **14**:926–937. https://doi.org/10.1111/pbi.12446.

Lu, S., Fadlalla, T., Tang, S., Li, L., Ali, U., Li, Q., and Guo, L. (2019). Genome-wide analysis of phospholipase D gene family and profiling of phospholipids under abiotic stresses in Brassica napus. Plant Cell Physiol. **60**:1556–1566. https://doi.org/10.1093/pcp/pcz071.

Ma, C., Zhang, Q., Lv, J., Qiao, K., Fan, S., Ma, Q., and Zhang, C. (2021). Genome-wide analysis of the phospholipase D family in five cotton species, and potential role of GhPLD2 in fiber development and anther dehiscence. Front. Plant Sci. **12**:728025. https://doi.org/10.3389/fpls.2021.728025.

Ma, Y., Wei, M., Zhang, T., Wang, Y., Duan, P., Wang, L., Kong, W., Zhang, G., and Wei, B. (2024). Functional analysis of the CaPIPLC5 gene in the regulation of the fertility restoration in pepper. Physiol. Plantarum **176**:e14429. https://doi.org/10.1111/ppl.14429.

McMahon, H.T., and Boucrot, E. (2015). Membrane curvature at a glance. J. Cell Sci. **128**:1065–1070. https://doi.org/10.1242/jcs.114454.

Nakamura, Y. (2017). Plant phospholipid diversity: emerging functions in metabolism and protein–lipid interactions. Trends Plant Sci. **22**:1027–1040. https://doi.org/10.1016/j.tplants.2017.09.002.

Nakamura, Y., and Ngo, A.H. (2020). Non-specific phospholipase C (NPC): an emerging class of phospholipase C in plant growth and development. J. Plant Res. **133**:489–497. https://doi.org/10.1007/s10265-020-01199-8.

Nakamura, Y., Tsuchiya, M., and Ohta, H. (2007). Plastidic phosphatidic acid phosphatases identified in a distinct subfamily of lipid phosphate phosphatases with prokaryotic origin. J. Biol. Chem. **282**:29013–29021. https://doi.org/10.1074/jbc.m704385200.

Nakanishi, H., de los Santos, P., and Neiman, A.M. (2004). Positive and negative regulation of a SNARE protein by control of intracellular localization. Mol. Biol. Cell **15**:1802–1815. https://doi.org/10.1091/mbc.e03-11-0798.

Nguyen, V.C., and Nakamura, Y. (2023). Distinctly localized lipid phosphate phosphatases mediate endoplasmic reticulum glycerolipid metabolism in Arabidopsis. Plant Cell **35**:1548–1571. https://doi.org/10.1093/plcell/koad021.

Noack, L.C., and Jaillais, Y. (2020). Functions of anionic lipids in plants. Annu. Rev. Plant Biol. **71**:71–102. https://doi.org/10.1146/annurev-arplant-081519-035910.

Peters, C., KIM, S.C., Devaiah, S., Li, M., and Wang, X. (2014). Non-specific phospholipase C 5 and diacylglycerol promote lateral root development under mild salt stress in Arabidopsis. Plant Cell Environ. **37**:2002–2013. https://doi.org/10.1111/pce.12334.

Peters, C., Li, M., Narasimhan, R., Roth, M., Welti, R., and Wang, X. (2010). Nonspecific phospholipase C NPC4 promotes responses to abscisic acid and tolerance to hyperosmotic stress in Arabidopsis. Plant Cell **22**:2642–2659. https://doi.org/10.1105/tpc.109.071720.

Potocký, M., Pleskot, R., Pejchar, P., Vitale, N., Kost, B., and Žárský, V. (2014). Live-cell imaging of phosphatidic acid dynamics in pollen tubes visualized by S po20p-derived biosensor. New Phytol. **203**:483–494. https://doi.org/10.1111/nph.12814.

Qi, F., Li, J., Ai, Y., Shangguan, K., Li, P., Lin, F., and Liang, Y. (2024). DGK5β-derived phosphatidic acid regulates ROS production in plant immunity by stabilizing NADPH oxidase. Cell Host Microbe **32**:425–440.e7. https://doi.org/10.1016/j.chom.2024.01.011.

Qin, H., Wang, J., Zhou, J., Qiao, J., Li, Y., Quan, R., and Huang, R. (2023). Abscisic acid promotes auxin biosynthesis to inhibit primary root elongation in rice. Plant Physiol. **191**:1953–1967. https://doi.org/10.1093/plphys/kiac586.

Sato, N., and Awai, K. (2017). "Prokaryotic pathway" is not prokaryotic: noncyanobacterial origin of the chloroplast lipid biosynthetic pathway revealed by comprehensive phylogenomic analysis. Genome Biol. Evol. **9**:3162–3178. https://doi.org/10.1093/gbe/evx238.

Scandola, S., and Samuel, M.A. (2019). A flower-specific phospholipase D is a stigmatic compatibility factor targeted by the self-incompatibility response in Brassica napus. Curr. Biol. **29**:506–512.e4. https://doi.org/10.1016/j.cub.2018.12.037.

Scholz, P., Pejchar, P., Fernkorn, M., Škrabálková, E., Pleskot, R., Blersch, K., Munnik, T., Potocký, M., and Ischebeck, T. (2022). DIACYLGLYCEROL KINASE 5 regulates polar tip growth of tobacco pollen tubes. New Phytol. **233**:2185–2202. https://doi.org/10.1111/nph.17930.

Serrano, N., Pejchar, P., Soukupová, H., Hubálek, M., and Potocký, M. (2022). Comprehensive analysis of glycerolipid dynamics during tobacco pollen germination and pollen tube growth. Front. Plant Sci. **13**:1028311. https://doi.org/10.3389/fpls.2022.1028311.

Sha, G., Sun, P., Kong, X., Han, X., Sun, Q., Fouillen, L., Zhao, J., Li, Y., Yang, L., Wang, Y., et al. (2023). Genome editing of a rice CDP-DAG synthase confers multipathogen resistance. Nature **618**:1017–1023. https://doi.org/10.1038/s41586-023-06205-2.

Sharma, P., Lakra, N., Goyal, A., Ahlawat, Y.K., Zaid, A., and Siddique, K.H.M. (2023). Drought and heat stress mediated activation of lipid signaling in plants: a critical review. Front. Plant Sci. **14**:1216835. https://doi.org/10.3389/fpls.2023.1216835.

Song, P., Jia, Q., Chen, L., Jin, X., Xiao, X., Li, L., Chen, H., Qu, Y., Su, Y., Zhang, W., and Zhang, Q. (2020). Involvement of Arabidopsis phospholipase D δ in regulation of ROS-mediated microtubule organization and stomatal movement upon heat shock. J. Exp. Bot. **71**:6555–6570. https://doi.org/10.1093/jxb/eraa359.

Stein, K.K., Primakoff, P., and Myles, D. (2004). Sperm-egg fusion: events at the plasma membrane. J. Cell Sci. **117**:6269–6274. https://doi.org/10.1242/jcs.01598.

Su, W., Raza, A., Gao, A., Zeng, L., Lv, Y., Ding, X., Cheng, Y., and Zou, X. (2023). Plant lipid phosphate phosphatases: current advances and future outlooks. Crit. Rev. Biotechnol. **43**:384–392. https://doi.org/10.1080/07388551.2022.2032588.

Su, Y., Li, M., Guo, L., and Wang, X. (2018). Different effects of phospholipase Dζ2 and non-specific phospholipase C4 on lipid remodeling and root hair growth in Arabidopsis response to phosphate deficiency. Plant J. **94**:315–326. https://doi.org/10.1111/tpj.13858.

Sugi, N., Calhau, A.R.M., Jacquier, N.M.A., Millan-Blanquez, M., Becker, J.D., Begcy, K., Berger, F., Bousquet-Antonelli, C., Bouyer, D., Cai, G., et al. (2024). The peri-germ cell membrane: poorly characterized but key interface for plant reproduction. Nat. Plants **10**:1607–1609. https://doi.org/10.1038/s41477-024-01818-5.

Sun, G., Geng, S., Zhang, H., Jia, M., Wang, Z., Deng, Z., Tao, S., Liao, R., Wang, F., Kong, X., et al. (2022). Matrilineal empowers wheat pollen with haploid induction potency by triggering postmitosis reactive oxygen species activity. New Phytol. **233**:2405–2414. https://doi.org/10.1111/nph.17963.

Tan, W.J., Yang, Y.C., Zhou, Y., Huang, L.P., Xu, L., Chen, Q.F., Yu, L.J., and Xiao, S. (2018). DIACYLGLYCEROL ACYLTRANSFERASE and DIACYLGLYCEROL KINASE modulate triacylglycerol and phosphatidic acid production in the plant response to freezing stress. Plant Physiol. **177**:1303–1318. https://doi.org/10.1104/pp.18.00402.

Tang, F., Xiao, Z., Sun, F., Shen, S., Chen, S., Chen, R., Zhu, M., Zhang, Q., Du, H., Lu, K., et al. (2020). Genome-wide identification and comparative analysis of diacylglycerol kinase (DGK) gene family and their expression profiling in Brassica napus under abiotic stress. BMC Plant Biol. **20**:473. https://doi.org/10.1186/s12870-020-02691-y.

Tei, R., and Baskin, J.M. (2022). Click chemistry and optogenetic approaches to visualize and manipulate phosphatidic acid signaling. J. Biol. Chem. **298**:101810. https://doi.org/10.1016/j.jbc.2022.101810.

Thole, J.M., Beisner, E.R., Liu, J., Venkova, S.V., and Strader, L.C. (2014). Abscisic acid regulates root elongation through the activities of auxin and ethylene in Arabidopsis thaliana. G3 (Bethesda) **4**:1259–1274. https://doi.org/10.1534/g3.114.011080.

Vaz Dias, F., Serrazina, S., Vitorino, M., Marchese, D., Heilmann, I., Godinho, M., Rodrigues, M., and Malhó, R. (2019). A role for diacylglycerol kinase 4 in signalling crosstalk during Arabidopsis pollen tube growth. New Phytol. **222**:1434–1446. https://doi.org/10.1111/nph.15674.

Waidmann, S., Sarkel, E., and Kleine-Vehn, J. (2020). Same same, but different: growth responses of primary and lateral roots. J. Exp. Bot. **71**:2397–2411. https://doi.org/10.1093/jxb/eraa027.

Wang, L., Liu, Y., and Hou, S. (2024a). DGK5 phosphorylation finetunes PA homeostasis in plant immunity. Trends Plant Sci. **29**:842–844. https://doi.org/10.1016/j.tplants.2024.03.017.

Wang, N.N., Ni, P., Wei, Y.L., Hu, R., Li, Y., Li, X.B., and Zheng, Y. (2024b). Phosphatidic acid interacts with an HD-ZIP transcription factor GhHOX4 to influence its function in fiber elongation of cotton (Gossypium hirsutum). Plant J. **118**:423–436. https://doi.org/10.1111/tpj.16616.

Wang, P., Shen, L., Guo, J., Jing, W., Qu, Y., Li, W., Bi, R., Xuan, W., Zhang, Q., and Zhang, W. (2019). Phosphatidic acid directly regulates PINOID-dependent phosphorylation and activation of the PIN-FORMED2 auxin efflux transporter in response to salt stress. Plant Cell **31**:250–271. https://doi.org/10.1105/tpc.18.00528.

Wang, X., Devaiah, S.P., Zhang, W., and Welti, R. (2006). Signaling functions of phosphatidic acid. Prog. Lipid Res. **45**:250–278. https://doi.org/10.1016/j.plipres.2006.01.005.

Wei, F., Fanella, B., Guo, L., and Wang, X. (2016). Membrane glycerolipidome of soybean root hairs and its response to nitrogen and phosphate availability. Sci. Rep. **6**:36172. https://doi.org/10.1038/srep36172.

Welti, R., Li, W., Li, M., Sang, Y., Biesiada, H., Zhou, H.E., Rajashekar, C.B., Williams, T.D., and Wang, X. (2002). Profiling membrane lipids in plant stress responses. Role of phospholipase D alpha in freezing-induced lipid changes in Arabidopsis. J. Biol. Chem. **277**:31994–32002. https://doi.org/10.1074/jbc.m205375200.

Wong, A., Donaldson, L., Portes, M.T., Eppinger, J., Feijó, J.A., and Gehring, C. (2020). Arabidopsis DIACYLGLYCEROL KINASE4 is involved in nitric oxide-dependent pollen tube guidance and fertilization. Development **147**:dev183715. https://doi.org/10.1242/dev.183715.

Yang, B., Li, M., Phillips, A., Li, L., Ali, U., Li, Q., Lu, S., Hong, Y., Wang, X., and Guo, L. (2021). Nonspecific phospholipase C4 hydrolyzes phosphosphingolipids and sustains plant root growth during phosphate deficiency. Plant Cell **33**:766–780. https://doi.org/10.1093/plcell/koaa054.

Yang, B., Li, J., Yan, J., Zhang, K., Ouyang, Z., Lu, Y., Wei, H., Li, Q., Yao, X., Lu, S., et al. (2023). Non-specific phospholipase C4 hydrolyzes phosphosphingolipids and phosphoglycerolipids and promotes rapeseed growth and yield. J. Integr. Plant Biol. **65**:2421–2436. https://doi.org/10.1111/jipb.13560.

Yang, B., Tan, Z., Yan, J., Zhang, K., Ouyang, Z., Fan, R., Lu, Y., Zhang, Y., Yao, X., Zhao, H., et al. (2024). Phospholipase-mediated phosphate recycling during plant leaf senescence. Genome Biol. **25**:199. https://doi.org/10.1186/s13059-024-03348-x.

Yao, H., Wang, G., Guo, L., and Wang, X. (2013). Phosphatidic acid interacts with a MYB transcription factor and regulates its nuclear localization and function in Arabidopsis. Plant Cell **25**:5030–5042. https://doi.org/10.1105/tpc.113.120162.

Yao, L., Zhang, Y., Liu, C., Liu, Y., Wang, Y., Liang, D., Liu, J., Sahoo, G., and Kelliher, T. (2018). OsMATL mutation induces haploid seed formation in indica rice. Nat. Plants **4**:530–533. https://doi.org/10.1038/s41477-018-0193-y.

Yao, S., and Wang, X. (2023). Monitoring lipid-protein interactions in planta using Förster resonance energy transfer. Methods Enzymol. **683**:243–252. https://doi.org/10.1016/bs.mie.2022.08.040.

Yao, S., Wang, G., and Wang, X. (2022a). Effects of phospholipase dε overexpression on soybean response to nitrogen and nodulation. Front. Plant Sci. **13**:852923. https://doi.org/10.3389/fpls.2022.852923.

Yao, S., Peng, S., and Wang, X. (2022b). Phospholipase Dε interacts with autophagy-related protein 8 and promotes autophagy in Arabidopsis response to nitrogen deficiency. Plant J. **109**:1519–1534. https://doi.org/10.1111/tpj.15649.

Yao, S., Kim, S.-C., Li, J., Tang, S., and Wang, X. (2024). Phosphatidic acid signaling and function in nuclei. Prog. Lipid Res. **93**:101267. https://doi.org/10.1016/j.plipres.2023.101267.

Yuan, S., Kim, S.C., Deng, X., Hong, Y., and Wang, X. (2019). Diacylglycerol kinase and associated lipid mediators modulate rice root architecture. New Phytol. **223**:261–276. https://doi.org/10.1111/nph.15801.

Zhang, Q., Lin, F., Mao, T., Nie, J., Yan, M., Yuan, M., and Zhang, W. (2012). Phosphatidic acid regulates microtubule organization by interacting with MAP65-1 in response to salt stress in Arabidopsis. Plant Cell **24**:4555–4576. https://doi.org/10.1105/tpc.112.104182.

Zhang, Q., Boundjou, N.B., Jia, L., Wang, X., Zhou, L., Peisker, H., Li, Q., Guo, L., Dörmann, P., Lyu, D., and Zhou, Y. (2023). Cytidine diphosphate diacylglycerol synthase is essential for mitochondrial structure and energy production in Arabidopsis thaliana. Plant J. **114**:338–354. https://doi.org/10.1111/tpj.16139.

Zhang, W., Wang, C., Qin, C., Wood, T., Olafsdottir, G., Welti, R., and Wang, X. (2003). The Oleate-Stimulated Phospholipase D, PLDδ, and

Phosphatidic Acid Decrease H2O2-Induced Cell Death in Arabidopsis. Plant Cell **15**:2285–2295. https://doi.org/10.1105/tpc.013961.

Zhang, Y., Zhu, H., Zhang, Q., Li, M., Yan, M., Wang, R., Wang, L., Welti, R., Zhang, W., and Wang, X. (2009). Phospholipase Dα1 and Phosphatidic Acid Regulate NADPH Oxidase Activity and Production of Reactive Oxygen Species in ABA-Mediated Stomatal Closure in Arabidopsis. Plant Cell **21**:2357–2377. https://doi.org/10.1105/tpc.108.062992.

Zhao, X., Xu, X., Xie, H., Chen, S., and Jin, W. (2013). Fertilization and Uniparental Chromosome Elimination during Crosses with Maize Haploid Inducers. Plant Physiol. **163**:721–731. https://doi.org/10.1104/pp.113.223982.

Zhong, Y., Chen, B., Li, M., Wang, D., Jiao, Y., Qi, X., Wang, M., Liu, Z., Chen, C., Wang, Y., et al. (2020). A DMP-triggered in vivo maternal haploid induction system in the dicotyledonous Arabidopsis. Nat. Plants **6**:466–472. https://doi.org/10.1038/s41477-020-0658-7.

Zhukovsky, M.A., Filograna, A., Luini, A., Corda, D., and Valente, C. (2019). Phosphatidic acid in membrane rearrangements. FEBS Lett. **593**:2428–2451. https://doi.org/10.1002/1873-3468.13563.

Plant Communications, Volume *6*

Supplemental information

Phosphatidic acid signaling in modulating plant reproduction and architecture

Shuaibing Yao, Bao Yang, Jianwu Li, Shan Tang, Shaohua Tang, Sang-Chul Kim, and Xuemin Wang

Supplemental Table 1. Accession numbers of genes encoding PA production and removal enzymes in Arabidopsis

Gene Family	Gene name	Accession number
Phospholipase D	PLDα1	AT3G15730
	PLDα2	AT1G52570
	PLDα3	AT5G25370
	PLDε	AT1G55180
	PLDβ1	AT2G42010
	PLDβ2	AT4G00240
	PLDγ1	AT4G11850
	PLDγ2	AT4G11830
	PLDγ3	AT4G11840
	PLDδ	AT4G35790
	PLDζ1	AT3G16785
	PLDζ2	AT3G05630
Diacylglycerol kinase	DGK1	AT5G07920
	DGK2	AT5G63770
	DGK3	AT2G18730
	DGK4	AT5G57690
	DGK5	AT2G20900
	DGK6	AT4G28130
	DGK7	AT4G30340
Lysophosphatidic acid acyltransferase	LPAT1	AT4G30580
	LPAT2	AT3G57650
	LPAT3	AT1G51260
	LPAT4	AT1G75020
	LPAT5	AT3G18850
Non-specific phospholipase C	NPC1	AT1G07230
	NPC2	AT2G26870
	NPC3	AT3G03520
	NPC4	AT3G03530
	NPC5	AT3G03540
	NPC6	AT3G48610
Phosphatidylinositol-hydrolyzing phospholipase C	PLC1	AT5G58670
	PLC2	AT3G08510
	PLC3	AT4G38530
	PLC4	AT5G58700
	PLC5	AT5G58690
	PLC7	AT3G55940
	PLC8	AT3G47290
	PLC9	AT3G47220

Phosphatidic acid phosphohydrolase	PAH1	AT3G09560
	PAH2	AT5G42870
Lipid phosphate phosphatase	LPP1	AT2G01180
	LPP2	AT1G15080
	LPP3	AT3G02600
	LPP4	AT3G18220
	LPPβ	AT4G22550
	LPPγ	AT5G03080
	LPPδ	AT3G58490
	LPPε1	AT3G50920
	LPPε2	AT5G66450
Cytidine diphosphate-diacylglycerol synthase	CDS1	AT1G62430
	CDS2	AT4G22340
	CDS3	AT4G26770
	CDS4	AT2G45150
	CDS5	AT3G60620
	CDS6	AT3G47630
